# The Dispenser's Column

**Published:** 1888-08-04

**Authors:** 


### The Dispenser's Column.

Petroleum oil is in cost nearly the same as gas for a light of equal intensity, but those who use it, unless with the best quality of lamps, rarely aim at having an equivalent illumination. Except with the purest varieties, its waste products are the same as those of gas, and it has the disadvantage that unless great care be taken in the trimming of the lamp, keeping it clean, and having it properly lighted, an unpleasant smell is caused.

Orosnes, the root of *Stachys affinis*, much in use in Japan as an article of diet, is coming into use in France and Belgium. The esculent portion consists of small root-stems of about the thickness of a finger. The flavour is said to be not strongly marked, but agreeable, recalling at once the artichoke, the potato, and salsify. The plant is very hardy, flourishes in poor soils, and bears the severest weather. The crop is fit to gather in November.

